# Editorial: Cities, violence and gender: Findings and concepts of the 21st century

**DOI:** 10.3389/fsoc.2022.1009483

**Published:** 2022-09-14

**Authors:** Anelise Gregis Estivalet, Gabriel Dvoskin

**Affiliations:** ^1^Stanford University, Stanford, CA, United States; ^2^University of Buenos Aires, Buenos Aires, Argentina

**Keywords:** violence, social movements, gender, sexual education, public policy

The city has always been a laboratory in which society experiences the dramas and challenges of its cohesion and experiments with ways of overcoming threats to its sustainable continuity. In recent decades, different types of violence, hitherto submerged, have become visible, altering the conceptual universe of violence. Until then, subordinate groups such as women, sexual dissent, and ethnic minorities managed to increase their discontent with their subordinate place in contemporary societies in the wider public domain. The social actions of groups concerned about gender violence or violence unleashed against minorities contributed to the idea that the visibility of violence is becoming a significant concept.

The Research Topic “*Cities, violence and gender: findings and concepts of the 21st century*” brings together critical contributions from various social actors, as well as problematizes public policies and relevant legislation to combat gender violence in different spheres of society. From innovative theories and methodologies, belonging to different disciplinary fields, the articles that make up this issue address issues of different types of corpus (social, political, cultural, legislative, journalistic, and media) and in different geographical spaces, from America to Africa, configuring an interdisciplinary and transnational perspective on these issues. Consequently, this Research Topic is intended to provide an up-to-date overview of a complex—still—conflicting society, as well as the points on which it is necessary to focus to promote the human rights of different social groups, such as peripheral populations, women, and people LGBTQIA+.

The articles focused on themes that involved gender, violence, and social movements. The main themes that emerged were inclusive/neutral language, sex education, social discourses, LGBTQIA+ population, women's activism, and agency. The issues addressed focused on discussions about: neutral and inclusive language and the importance of language education for society (Bonnin and Coronel; Tosi); socio-discursive representations and social discourses on topics such as abortion, sex workers and transgender sex workers, transgender women and LGBTQIA+ populations (Bagagli et al.; Benner; Dvoskin; Ribeiro et al.; Soich); and, on gender and women's activism (Moura and Cerdeira; Vieira and Rocha; Estivalet).

Finally, the articles analyzed the need to broaden debates on topics that were hitherto considered controversial, but which have increasingly become relevant to public debate, contributing to the improvement of education, to changing attitudes and behaviors, for the reduction of gender inequalities, violence, and prejudice, in search of a more just, egalitarian society that respects differences.

We are grateful to all researchers who shared their valuable contributions, believing that this Research Topic can serve as inspiration and support for academics, practitioners, and readers working on gender issues and as a starting point for generating interdisciplinary links at the southern and north levels global, helping to generate more just and egalitarian societies in terms of gender.

## Author contributions

All authors listed have made a substantial, direct, and intellectual contribution to the work and approved it for publication.

## Conflict of interest

The authors declare that the research was conducted in the absence of any commercial or financial relationships that could be construed as a potential conflict of interest.

## Publisher's note

All claims expressed in this article are solely those of the authors and do not necessarily represent those of their affiliated organizations, or those of the publisher, the editors and the reviewers. Any product that may be evaluated in this article, or claim that may be made by its manufacturer, is not guaranteed or endorsed by the publisher.

